# Effects of one-lung flooding on porcine haemodynamics and gas exchange

**DOI:** 10.7150/ijms.50852

**Published:** 2020-10-23

**Authors:** Thomas Lesser, Frank Wolfram, Conny Braun, Reiner Gottschall

**Affiliations:** 1 Department Thoracic and Vascular Surgery, SRH Wald-Klinikum Gera, Teaching Hospital of Jena University Hospital, Strasse des Friedens 122, Gera D-07548, Germany.; 2 Central Experimental Animal Facility, University Hospital Jena, Location Dornburger Strasse 23a, Jena D-07743, Germany.; 3 Doctor Emeritus, Department of Anaesthesiology and Intensive Care, Jena University Hospital, Am Klinikum 1, Jena D-07747, Germany.

**Keywords:** orcine model of one-lung flooding, haemodynamics, gas exchange

## Abstract

**Background and aim:** We established a porcine model of one-lung flooding (OLF) that can be used for research on the use of ultrasound for lung tumour detection, ultrasound-guided transthoracic needle biopsy, and tumour ablation. However, OLF requires one-lung ventilation (OLV) and eliminates the recruitment strategies of the nonventilated lung. During thoracic surgery, OLV alone can be associated with hypoxia, hypercapnia, and right ventricular overload. Here, we examined whether OLF influences haemodynamics and gas exchange indices during and after OLV/OLF compared with OLV/apnoea and two-lung ventilation (TLV) following deflooding.

**Methods:** Fourteen pigs were included in this study: five were allocated to the control group (CO) and nine were assigned to the OLF group (OLF). Assessments of haemodynamics, gas exchange, and lung sonography were performed after baseline measurements, during OLV/apnoea, OLV/OLF, and after deflooding and TLV. The volume of extravascular lung water was also measured.

**Results:** OLF induced no significant deterioration of oxygenation or ventilation during OLF or after deflooding and TLV. Color-coded duplex sonography of the pulmonary artery in the flooded lung demonstrated an oscillating flow that corresponded to intrapulmonary circulatory arrest. After flooding of the nonventilated lung, the partial pressure of O_2_ in the arterial blood increased and the shunt fraction decreased significantly compared to OLV/apnoea conditions. After deflooding and TLV, haemodynamics and gas exchange indices showed no differences compared to the CO group and baseline values, respectively.

**Conclusions:** OLF is safe to use during acute animal experimentation. No clinically relevant deterioration of haemodynamics or gas exchange occurred during or after OLF. Due to the circulatory arrest in the flooded lung, the right-to-left shunt volume in the nonventilated lung was minimized. Survival experiments are necessary to further assess the utility of this method.

## Introduction

One-lung flooding (OLF) was developed to enable complete lung sonography of the flooded lung 1,2. This novel method provides new diagnostic and therapeutic options for clarifying small pulmonary nodules and for performing focused ultrasound ablation of lung tumours. Thus, it could make the diagnosis and treatment of human lung tumours more efficient and less invasive 3-5. OLF requires one-lung ventilation (OLV) and eliminates the recruitment strategies of the nonventilated lung. OLV alone (apnoea of the nonventilated lung, OLV/apnoea) is associated with many problems such as hypoxia, hypercapnia or right ventricular overload 6-8. Many recruitment strategies, such as the application of continuous positive airway pressure (CPAP), high-frequency jet ventilation (HFJV), and intermitted manual ventilation of the nonventilated lung, are commonly used during thoracic surgery to avoid threatening disturbances in gas exchange when using OLV alone 9-13. These strategies are impossible to implement during OLV/OLF.

The effects of OLV/OLF on haemodynamics and gas exchange with closed thorax have not been studied to date. OLF after atelectasis of the nonventilated lung following thoracotomy or thoracoscopy has a positive influence on oxygenation and shunt fraction 14,15. However, the chest opening should be avoided in future clinical use because ultrasound-guided interventions such as focused ultrasound ablation of lung tumours are possible transcutaneously. In addition, later studies showed that atelectasis of the lung to be flooded is also not necessary 2. Only a few studies of gas exchange during whole-lung lavage exist, which describe a therapeutic method to physically remove proteinaceous materials from the alveolar space. The highest level of oxygen saturation is usually seen during the completion of the filling phase, whereas the oxygen level drops as the lavaged lung is emptied 16-18.

Prior to the implementation of OLF on humans, the safety of the method must be studied. Pigs are commonly used as animal models of the human respiratory and cardiovascular systems because of their comparable physiology to that of humans 19-21. Many porcine experimental studies have investigated haemodynamics, oxygenation, and pulmonary shunt fraction and perfusion during OLV 22-24. In this study, we examined whether OLF influences the haemodynamics and gas exchange indices during and after OLV/OLF compared with OLV/apnoea and two-lung ventilation (TLV).

## Materials and Methods

After approval provided by the Veterinary Department of the Thuringian State Authority for Food Protection and Fair Trading (TLLV Reg. 22-2684-04-WKG-16-002), this study was performed in laboratories at the Central Experimental Animal Facility, University Hospital Jena. All animals were kept in groups and housed for 4 days prior to the study and were thus acclimated to their new surroundings. All procedures were performed in compliance with the National Animal Protection Act.

### Animal preparation

Fourteen juvenile female pigs (German Landrace, mean age 12.4 weeks), weighing an average of 37 kg (range: 35-40 kg), were included in this study. Before the experiments began, the health status of each animal was assessed by a veterinarian.

Ketamine (25 mg/kg) and midazolam (0.2 mg/kg) were administered intramuscularly as premedicants to each pig. General anaesthesia was induced by the administration of propofol (3 mg/kg) and fentanyl (2.7 µg/kg) via a peripheral vein and maintained with a continuous infusion of propofol (6 mg/kg/h) and fentanyl (2.7 µg/kg) every hour. After the onset of anaesthesia, the pigs were intubated transorally using a single lumen endotracheal tube (6.5 ID, Dahlhausen, Köln, Germany). Pancuronium bromide, (0.06 mg/kg, IV) was administered every hour as a muscle relaxant, and mechanical ventilation was performed using an ICU ventilator (Servo 900 C, Siemens AG, Munich, Germany) with pressure-controlled settings [fraction of inspired oxygen (FiO_2_): 0.4; target tidal volume: 10 mL/kg; inspiratory to expiratory ratio: 1:1.9; respiratory rate: 20 breaths/min; and positive end-expiratory pressure: 4 cm H_2_O]. These ventilator settings were maintained throughout the experiment (optimized based on an end-tidal CO_2_ range of 35-45 mmHg), except for FiO_2_, which was increased to 1.0 at the time of blood sample collection for blood gas analysis (to determine the pulmonary shunt fraction) and for denitrogenating of the lung before OLF.

A sterile technique was used to place an arterial catheter (Arterial Leader Cath 2.7 Fr; Vygon, Ecouen, France), which was advanced 10 cm into position in the central common carotid artery for haemodynamic monitoring and arterial blood gas sampling. A flow-directed pulmonary artery catheter (6 Fr, Swan Ganz, Edwards Lifesciences, Irvine, CA, USA) was inserted through an 8.0 Fr introducer sheath (Arrow International, Reading, PA, USA) that was placed into the right internal jugular vein. Then, a left-sided, double lumen endobronchial tube (35 Fr, DLT), which was designed for use in pigs and specifically made for this study (Medicoplast, International GmbH, Illingen, Germany), was placed with the pig in the supine position via an airway exchange catheter (11.0 Fr, 100 cm, extra-firm with a soft tip, COOK Deutschland GmbH, Mönchengladbach, Germany). The correct position of the tube was confirmed by fibreoptic bronchoscopy (BF 3C30, Olympus, Tokyo, Japan). A cuff controller (VBM Medizintechnik GmbH, Sulz, Germany) was used to maintain constant cuff pressures (50 cm H_2_O for both endobronchial and tracheal cuffs).

All animals were connected to a Datex monitor (Datex AS3 Monitoring System, Datex-Ohmeda Corp., Helsinki, Finland) for continuous vital data assessment (haemodynamic and respiratory parameters, including intrapulmonary pressure). All blood gas measurements were analysed using a blood gas analyser (Rapidpoint 405, Siemens Healthcare, Erlangen, Germany), and the output was used to calculate the pulmonary shunt fraction.

### Experimental protocol

The experimental (OLF) and control (CO) groups were studied successively. After initial preparations, baseline measurements were performed following a 30-minute period of stabilisation under TLV with the animals in the supine position. Thereafter, the left bronchial lumen of the DLT was disconnected from the ventilator to perform one-lung ventilation with apnoea of the nonventilated lung (OLV/apnoea) for 15 minutes under a FiO_2_ of 1.0. Then, both lungs were continuously ventilated with a FiO_2_ of 1.0 for 20 minutes to enable the denitrogenation of the lung as a requirement for complete OLF.

**Figure 1:**
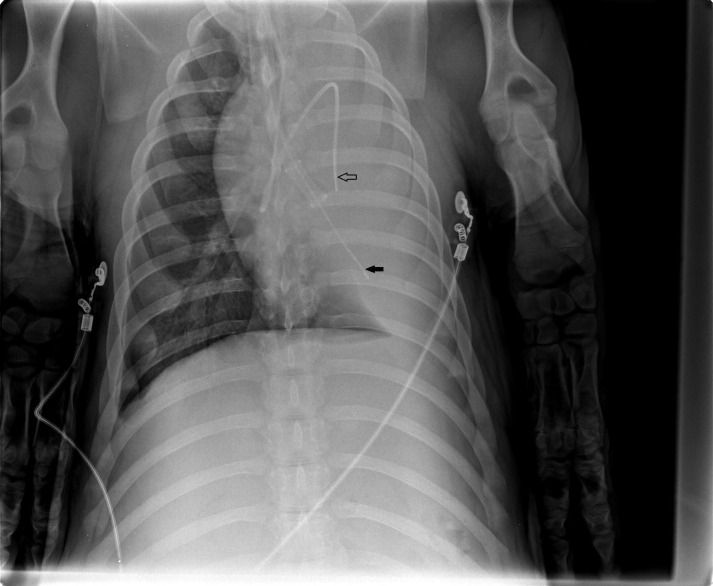
Chest X-ray after one-lung flooding of the left lung, showing the pulmonary artery catheter in the left main artery (arrow) and the intrapulmonary catheter via the left bronchial lumen of the DLT (fat arrow).

The animals were placed in the left lateral decubitus position (the lung to be flooded down), and OLF performed as follows: The left bronchial lumen of the DLT was disconnected from the ventilator, and the infusion system was immediately connected to the left tube leg. The left lung was slowly filled (single filling) with degassed and warmed (37 °C) isotonic saline that flowed passively from an infusion bottle suspended 50 cm above heart level. The volume to be infused was estimated as one-half of the functional residual capacity of the lung (12.5 mL/kg). To prevent barotrauma and bubble formation, the saline volume was infused no faster than 125 mL/min (assuming a basal oxygen consumption of 250 mL/min 25). Complete saline filling was monitored using transcutaneous lung ultrasound. At the end of the filling process, the animals were returned to the supine position, and an intrapulmonary catheter (Fogarty, Tru-lumen Embolectomy Catheter, 5F, Edwards Lifesciences, Unterschleissheim , Germany) was introduced via the left bronchial lumen of the DLT using a multiport airway adapter to avoid fluid outflow (Arndt Endobronchial Blocker Set, Cook Medical Europe, Ireland). The position of the pulmonary artery catheter and the intrapulmonary catheter were documented using ultrasound and chest X-ray analyses (**Fig. 1**).

The status of OLV/OLF was maintained for 3 hours. Thereafter, the fluid was passively drained from the left lung by placing the animal in the Trendelenburg position followed by TLV over a 30-minute period (15 minutes in the right lateral position and 15 minutes in the supine position). The volume of the recovered fluid was then measured. In the CO group, after baseline measurements were obtained, all animals stayed in the supine position and ventilated (TLV) via DLT for 3 hours and 30 minutes.

Haemodynamics and blood gas analyses were monitored at the following time points: at baseline, 15 minutes after OLV/apnoea, 3 hours after OLV/OLF, and after 30 minutes of TLV following deflooding. In the CO group, measurements were performed temporally in accordance with the OLF group.

### Lung ultrasound and extravascular lung water (EVLW)

For sonographic examination, a sonographic system (Flex Focus 800, BK Medical, Arhus, Denmark) with linear (8870, 18-6 MHz) and curved arrays (8815, 10-4 MHz) were used. In all animals following OLF, the central pulmonary artery within the flooded lung was located using transthoracic ultrasound, considering the same probe position on the lateral chest wall. The angle adjusted (<60°) Doppler was positioned in the pulmonary artery, and a sample volume was chosen to cover at least one-half of the entire flow width. The flow curves were recorded hourly during flooding. Comparable duplex sonography of the pulmonary arteries in the right nonflooded lung was impossible due to gas in the ventilated lung.

Transthoracic lung sonography was used to determine pleural effusion and lung water content for both lungs at the end of the 30 minutes of TLV following deflooding. To evaluate the lung water content, B-line and pleural line changes were recorded.

Following euthanasia, the thoracic cavity was carefully opened, and the heart and lungs were harvested. The heart was removed so blood could be drained from the lungs. To measure the EVLW, representative tissue samples were taken from the lobes of both lungs and immediately weighed to obtain the wet weight. The samples were weighed again after 24 hours of drying in an oven at 70 °C. The wet-to-dry ratio was calculated as follows: wet/dry [%] = (weight_wet_ - weight_dry_)/weight_wet_ × 100.

### Statistical methods

All data were analysed using MedCalc Statistical Software, version 19.1.7 (MedCalc Software bv, Ostend, Belgium), and distributions were confirmed using the Q-Q plot. Therefore, median values and interquartile ranges are presented. The Mann-Whitney U test was used to analyse differences between independent groups (i.e., OLF and CO groups). Statistical significance (two sided) was set at a *P* value less than 0.05. For analysis of changes between measurement points within each group, the Friedmann test followed by a post hoc test for *P* values both less than 0.05 and less than 0.01 were performed.

## Results

In total, 14 animals were examined in this study. Five animals served as controls, and nine animals were studied in the OLF group. One animal died of acute cardiac death as a result of difficult intubation prior to OLF. In one animal, a partial fluid run-over occurred due to DLT dislocation. The data from these animals were included from the beginning of the experiment until the last useful measurement. The DLT was placed again under fibrebronchoscopic control and the liquid was sucked off. The situation could be controlled without harm. No animals died due to OLF.

The baseline animal data showed no differences between groups. The systolic and mean pulmonary artery pressures (SPP and MPP) increased marginally in the OLF group compared to the CO group by 6 mmHg and 5 mmHg, respectively [SPP: 35 (24.3-36.0) mmHg vs. 29 (27.3-31.3) mmHg, *P* = 0.004; MPP: 28 (27.0-28.0) mmHg vs. 23 (20.8-26.5) mmHg, *P* = 0.02]. Heart rate, mean arterial pressure, and diastolic pulmonary artery pressure were unchanged during the entire study. During OLF, the intrapulmonary pressure in the flooded lung was 13 [9.5-14.5] mmHg (**Table 1**).

**Figure 2:**
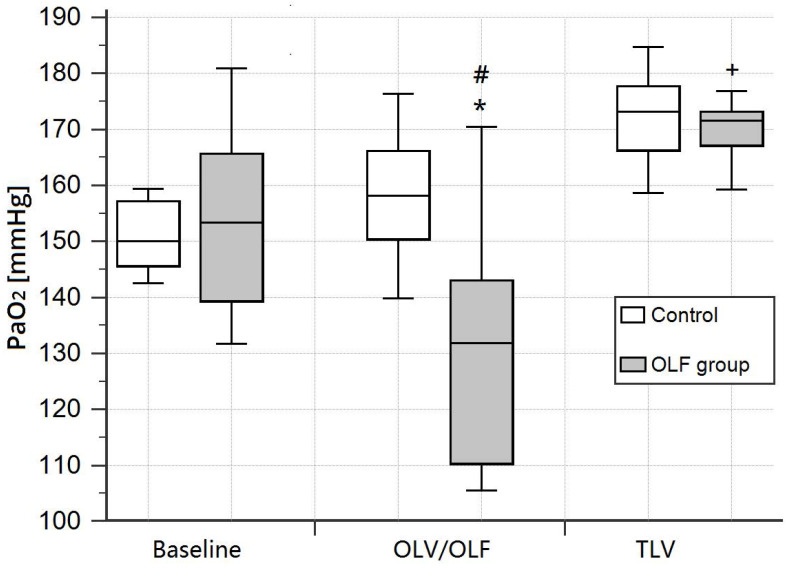
Arterial oxygen partial pressure (PaO_2_) at baseline, during one-lung ventilation/one-lung flooding (OLV/OLF), and after deflooding and two-lung ventilation (TLV). Abbreviations: PaO_2_: arterial oxygen partial pressure using an inspired fraction of oxygen of 0.4, OLV: one-lung ventilation, OLF: one-lung flooding, TLV: two-lung ventilation, CO group: control group, OLF group: OLF experimental group. *P < 0.05, significant compared with the CO group, #*P* < 0.01, significant compared with baseline and TLV values, +*P* < 0.05, significant compared with baseline values.

**Figure 3:**
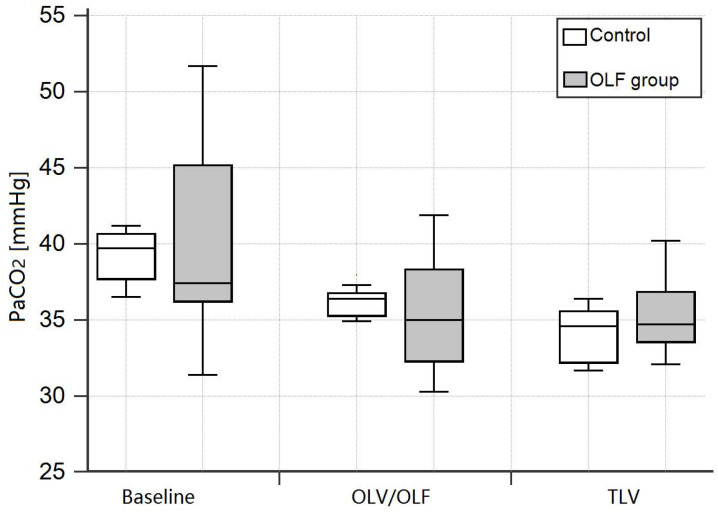
Arterial carbon dioxide partial pressure (PaCO_2_) at baseline, during one-lung ventilation/one-lung flooding (OLV/OLF), and after deflooding and two-lung ventilation (TLV). Abbreviations: PaCO_2_: arterial carbon dioxide partial pressure, OLV: one-lung ventilation, OLF: one-lung flooding, TLV: two-lung ventilation, CO group: control group, OLF group: OLF experimental group. Changes in the OLF group are non-significant.

The PaO_2_ (FIO_2_ = 0.4) during OLF was slightly decreased in the OLF group compared to the CO group [131.8 (110.3-143.0) mmHg vs. 158.2 (150.5-166.1) mmHg, *P* = 0.02] and baseline values [vs. 153.3 (139.4-165.6) mmHg, *P* < 0.01]. Arterial oxygen saturation (SaO_2_), arterial carbon dioxide partial pressure (PaCO_2_), and pH were unchanged in the OLF group compared to the CO group (**Fig. 2, 3**)).

**Figure 4:**
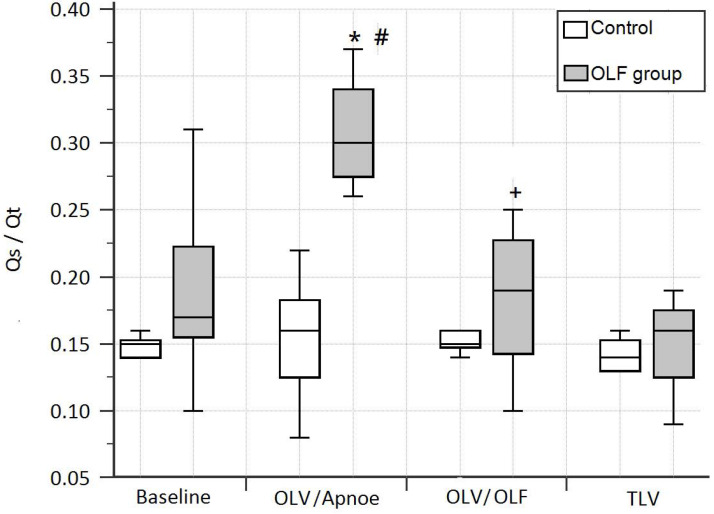
The shunt fraction (Qs/Qt) at baseline, during one-lung ventilation/apnoea (OLV/apnoea), during one-lung ventilation/one-lung flooding (OLV/OLF), and after deflooding and two-lung ventilation (TLV). OLF experimental group (grey); control group (white). *P < 0.05, significant compared with the CO group, #*P* < 0.01, significant compared with baseline values, +*P* < 0.01, significant compared with OLV/apnoea.

In the OLF group during OLV/OLF, the pulmonary right-to-left shunt fraction (Qs/Qt) was 4% higher than in the CO group, but this difference was not significant. In contrast to OLV/OLF, OLV/apnoea led to a significant decrease in the partial pressure of oxygen and an increase in the shunt fraction compared to the CO group [PaO_2_ (1.0): 205.5 (191.2-216.4) mmHg vs. 461.6 (451.7-478.7) mmHg, *P* = 0.01; Qs/Qt: 0.3 (0.28-0.34) vs. 0.16 (0.12-0.18), *P* = 0.01]. After flooding of the nonventilated lung, PaO_2_ increased and the shunt fraction decreased significantly compared to OLV/apnoea [PaO_2_ (1.0): 400.1 (338.1-464.7) vs. 205.5 (191.2-216.4) mmHg, *P* < 0.01; Qs/Qt: 0.19 (0.14-0.23) vs. 0.3 (0.28-0.34), *P* < 0.01]; **Table 2, Fig. 4**].

After deflooding and TLV, haemodynamics and gas exchange indices showed no differences compared to the CO group and baseline values, respectively.

**Figure 5:**
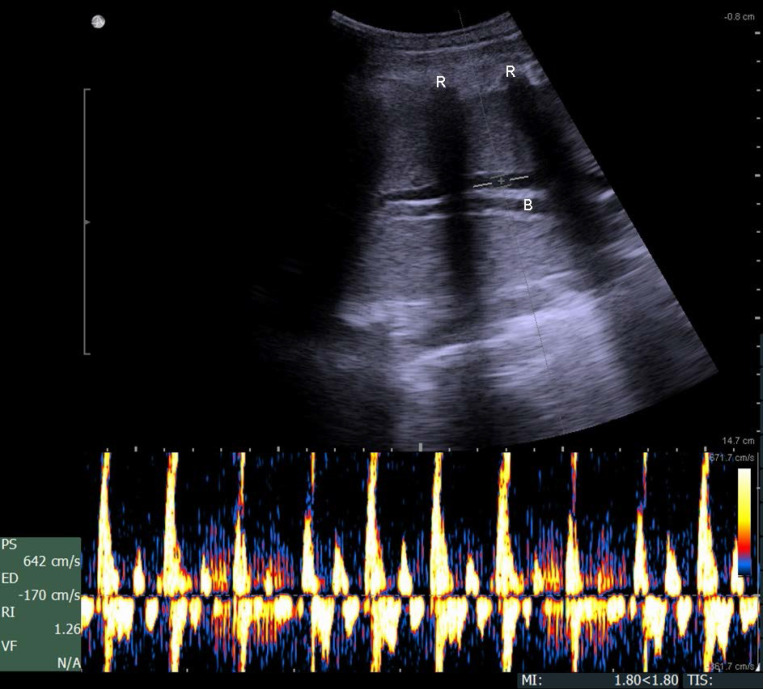
Duplex sonography imaging shows a biphasic oscillating flow in the pulmonary artery within the flooded lung (B, bronchus; R, ribs).

Color-coded duplex sonography of the pulmonary artery within the flooded lung revealed a strong oscillating flow pattern, which was found in all experiments (**Fig. 5**).

**Figure 6:**
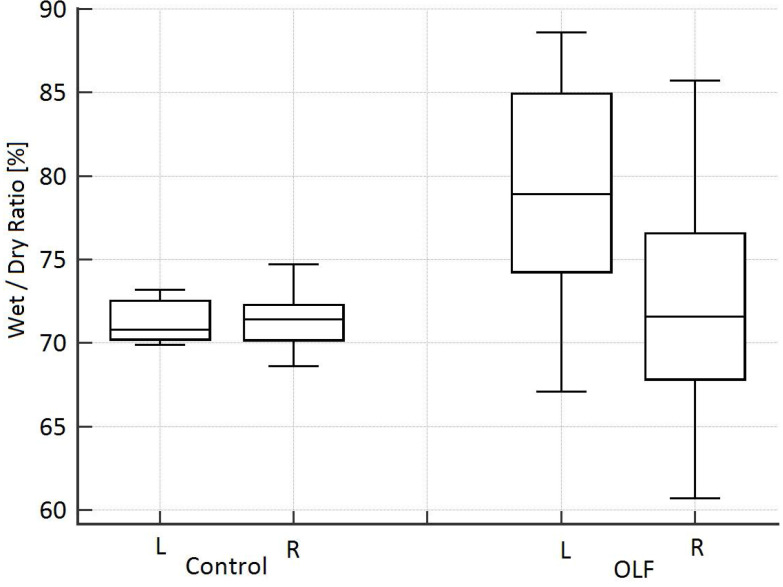
Wet/dry weight ratio in both lungs (L = left lung, flooded for 3 hours; R = right lung, only ventilated) 30 minutes after deflooding and two-lung ventilation in the one-lung flooding (OLF) group compared to the control group.

The amount of liquid recovered was 297.5 mL (281.8-310.0 mL), which corresponds to a recovery rate of 63.2% [saline filling amount: 475 mL (463-496 mL)]. Thirty minutes after deflooding and TLV, the EVLW volume was 11% higher in the flooded lung compared to the CO group [78.9% (75.5-84.3%) vs. 70.8% (70.3-72.3%)], but these differences were not significant (**Fig. 6**).

**Figure 7:**
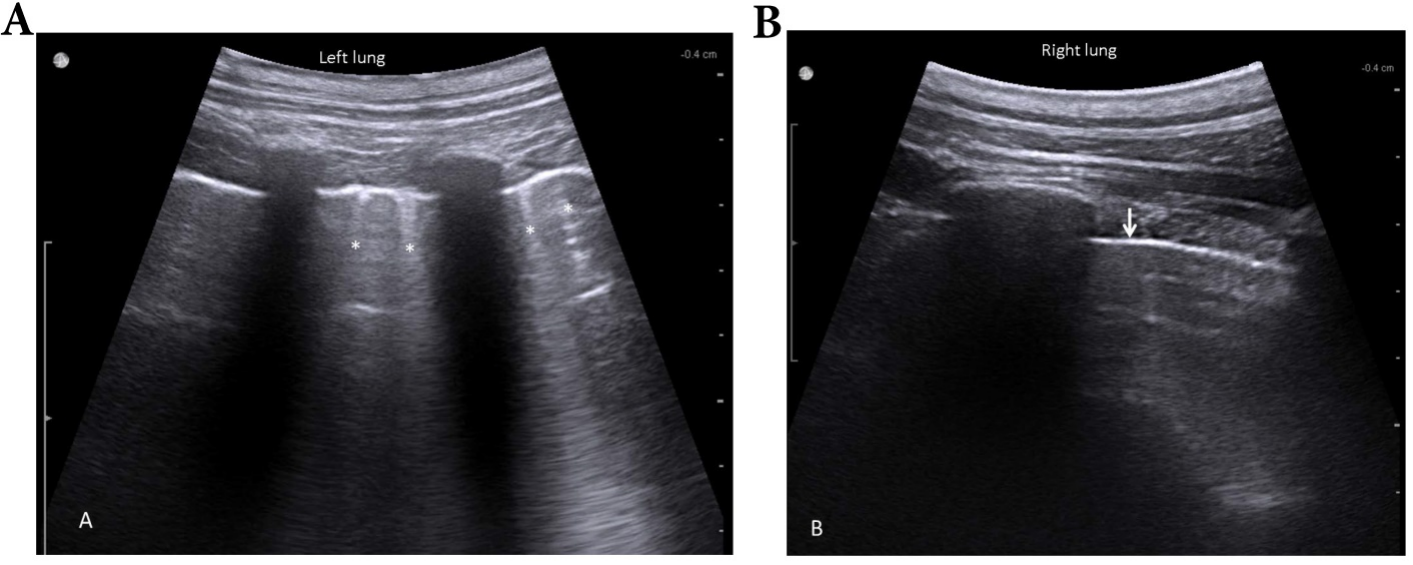
Lung sonography 30 minutes after deflooding and two-lung ventilation. Illustrated are the left lung (filled with saline over 3 hours, A) and the right lung (only ventilated, B). The left lung shows a slightly thickened pleural line and few B-lines (stars); the right lung presents a sharp pleural line (arrow) and no B-lines.

At this point, lung sonography showed few B-lines and a slight increase of pleura thickening in absence of subpleural consolidations or pleural effusions of the previously flooded lung compared to the right lung (**Fig. 7**).

## Discussion

In this study, the effects of OLF on haemodynamics and gas exchange with closed thorax were examined. The key result was that OLF did not influence haemodynamics or gas exchange during or after flooding in a clinically relevant manner. OLV and apnoea in the nonventilated lung caused a relevant decrease in PaO_2_ and an increase of shunt fraction, which are well-known problems during thoracic surgery using OLV without recruitment strategies of the nonventilated lung, such as CPAP or HFJV 7-9. In contrast, such deterioration of both oxygenation and ventilation did not occur under OLV/OLF. Previous studies showed comparable results under the conditions of thoracoscopy or thoracotomy 14,15. Thus, the conditions under which the OLF is performed (at opened or closed thorax) do not influence the hemodynamics and gas exchange. Saline filling of one lung with half of the functional residual capacity led to an intrapulmonary pressure of 13 mmHg, which is twice the capillary pressure. Our previous measurements for OLF after thoracotomy showed that the pulmonary capillary pressure (measured by a catheter in the left atrium) was 7 mmHg 26. The pulmonary capillary wedge pressure measurements in this study were not usable because of a false high pressure caused by the placement of the pulmonary artery catheter in the flooded lung. The transmural pressure difference, which includes a markedly higher intrapulmonary pressure, occludes the venous region of the capillary bed. Therefore, it can be assumed that pulmonary perfusion ceases in a large part of the flooded lung.

Although one-time unilateral lung flooding must be distinguished from bronchopulmonary whole-lung lavage, the physiologic alterations can be anticipated from the hemodynamic response of the pulmonary circulation to the variations in the airway pressure that occur as the airways are cyclically filled and emptied. The highest oxygen saturation level is usually seen at the completion of the filling phase when the blood is physiologically shunted from the nonventilated to the ventilated lung. Conversely the oxygen level will drop as the lavage lung is emptied 16.

The oscillating flow in the pulmonary artery of the flooded lung is supportive evidence of the arrest of the pulmonary circulation. Similarly, oscillating flow patterns recorded in the extracranial cerebral arteries of brain-dead patients are considered to be conclusive signs of cerebral circulatory arrest 27,28. The oscillating Doppler pattern is defined as a biphasic flow velocity spectrum with equivalent, opposing inflow and outflow components, such that the resulting time-averaged mean velocity in the evaluated vessel is zero 29.

The insignificant increase in the shunt fraction (4%) in the OLF group compared to the CO group could have different causes. First, both the intrapulmonary pressure and the right-to-left shunt volume are dependent on the filling volume. In previous experiments to determine the correct fill volume, we observed an increased shunt fraction when the fill volume was less than 12.5 mL/kg body mass. Second, in the nondependent regions of the flooded lung where the hydrostatic intrapulmonary pressure is below 7 mmHg, perfusion still occurs due to uniform blood flow 30. Third, due to redistribute blood from the flooded into the ventilated lung an increased perfusion/ventilation mismatch in the ventilated lung can occur, causing an increase in the shunt fraction in the ventilated lung.

Despite an approximate 50% reduction in the pulmonary artery cross-sectional area as a result of the flooding of one lung, both the SPP and MPP increased marginally by 6 mmHg and 5 mmHg, respectively, compared to the CO group. Both vascular compliance and capillary recruitment are mechanisms in the ventilated lung that can compensate for the increased vascular resistance in the flooded lung. Similarly, a considerable increase in cardiac output in healthy humans is accompanied only by a marginal increase in pulmonary arterial pressure 31.

After deflooding and TLV over 30 minutes, there is little residual fluid volume in the left lung compared to the right nonflooded lung. The EVLW was 11% higher than in the right lung. In a previous study we found a 6.2% higher EVLW in the flooded lung, however the flooding time was only 60 minutes 32. The described method to determine EVLW cannot differentiate between intraalveolar and interstitial fluid accumulation. But we found a slight increase of B-lines that indicate oedematous thickening of interlobular septa 33. This residual fluid does not negatively impact the gas exchange. PaO_2_, PaCO_2_, and the shunt fraction 30 minutes after TLV did not differ in the OLF group compared to the CO group and baseline values.

Our study has some limitations that should be addressed. First, the study was carried out on animals with healthy lungs. OLF in patients with chronic obstructive or interstitial lung disease could affect the haemodynamics and gas exchange indices. However, whole-lung lavage under OLV to treat pulmonary alveolar proteinosis, which causes severe hypoxemic respiratory failure, was considered safe. In one survey of 20 worldwide centres that perform whole-lung lavage in adults, the most common indications were declining lung function, declining oxygenation, and radiographic worsening 34. The second limitation was the use of the present calculation of the intrapulmonary right-to-left shunt volume because no differentiation can be made between the shunt volume percentages of the right and left lungs. We hypothesize that the 4% increase in the shunt fraction during OLF/OLV was caused by a ventilation/perfusion mismatch in the ventilated lung due to redistribute blood from the flooded into the ventilated lung. Only the determination of the side-separated shunt volume would help clarify this hypothesis. In a previous study, we described a novel intraoperative method to determine side-separated shunt volumes 26. A third limitation was that the investigations were carried out the procedure only with the animals in the supine position. There is a need to examine the haemodynamics and gas exchange indices with the animals in the lateral decubitus position, such as with the flooded lung in both nondependent and dependent positions, because therapeutic procedures, such as ultrasound- or magnetic resonance-guided focused ablation of lung tumours, require various positions. Finally, the study used an acute experimental model to collect data only up to 30 minutes after deflooding and TLV under general anaesthesia. Survival experiments must be performed where gas exchange indices are measured after extubation and spontaneous ventilation to get closer to future clinical practice.

## Conclusions

We examined whether haemodynamics and gas exchange parameters were influenced by OLV/OLF with closed thorax in a clinically relevant manner. We demonstrated that OLV/OLF influenced the parameters only slightly and maintained them in the normal physiological range. Due to circulatory arrest in the flooded lung, the right-to left shunt volume in the nonventilated lung was minimized. This finding implies that OLF overcomes the declines in oxygenation and ventilation that occur during OLV alone. Additionally, the residual fluid after deflooding and TLV over 30 minutes does not negatively impact the haemodynamics and gas exchange indices. These results should be verified in experiments measuring effects on survival.

## Figures and Tables

**Table 1:** Haemodynamics at baseline, during one-lung flooding and after deflooding and two-lung ventilation

Group	Baseline	OLV/OLF	TLV	*P* Value
**HR, bpm**				
CO group	96 (91.5-101.5)	92 (89.3-98.0)	94 (92.7-97.5)	0.54
OLF group	99(94.3-110.7)	95 (92.5-103.3)	91^#^ (87.5-100.5)	**0.02**
*P* value*	0.26	0.31	0.55	
**MAP, mmHg**				
CO group	79 (75.3-86.8)	75 (70.3-81.3)	81 (59.0-82.8)	0.85
OLF group	84 (80.3-96.0)	79 (63.0-89.8)	90 (79.8-101.3)	0.09
*P* value*	0.21	0.69	0.12	
**SSP, mmHg**				
CO group	29 (26.5-30.3)	29 (27.3-31.3)	28 (26.8-28.5)	0.41
OLF group	30 (29.0-31.5)	35^#^ (33.5-35.5)	32 (27.5-37.0)	**0.02**
*P* value*	0.2	**0.004**	0.16	
**DPP, mmHg**				
CO group	19 (16.8-20.3)	22 (18.5-23.3)	19 (17.8-21.5)	0.37
OLF group	21 (19.3-21.8)	21 (20.0-23.0)	22 (19.0-25.0)	0.56
*P* value*	0.19	0.9	0.17	
**MPP, mmHg**				
CO group	24 (21.8-25.3)	23 (20.8-26.5)	24 (23.8-25.3)	0.96
OLF group	25 (24.0-25.3)	28^+^ (27.0-28.0)	26^+^ (25.2-29.0)	**0.007**
*P* value*	0.16	**0.02**	**0.027**	
**IPP, mmHg**				
OLF group		13 (9.5-14.5)		

Abbreviations: HR: heart rate, MAP: mean artery pressure, SPP: systolic pulmonary artery pressure, DPP: diastolic pulmonary artery pressure, MPP: mean pulmonary artery pressure, IPP: intrapulmonary pressure, OLV: one-lung ventilation, OLF: one-lung flooding, TLV: two-lung ventilation, CO group: control group, OLF group: experimental OLF group; *P < 0.05, significant compared with the CO group, ^#^*P* < 0.05, significant compared with baseline values, ^+^*P* < 0.01, significant compared with baseline values.

**Table 2:** Arterial oxygen partial pressure and shunt fraction at baseline, during one-lung ventilation/apnoea, one-lung flooding, and after deflooding and two-lung ventilation

Characteristic		Baseline	OLV/apnoea	OLV/OLF	TLV	*P* Value
PaO_2_, mmHg	CO group	474.4 (463.4-486.0)	461.6 (451.7-478.7)	450 (442.4-465.0)	497.8 (442.3-515.3)	0.35
	OLF group	453.9 (438.8-494.2)	205.5^#^ (191.2-216.4)	400.1^+^ (338.1-464.7)	444.2 (400.3-470.7)	**0.0015**
	*P* value*	0.61	**0.01**	0.22	0.22	
Qs/Qt	CO group	0.15 (0.14-0.153)	0.16 (0.125-0.183)	0.15 (0.148-0.16)	0.14 (0.13-0.18)	0.6
	OLF group	0.17 (0.16-0.22)	0.3^#^ (0.28-0.34)	0.19^+^ (0.14-0.23)	0.16 (0.13-0.15)	**0.0035**
	*P* value*	0.077	**0.01**	0.22	0.27	

Abbreviations: PaO_2_: arterial oxygen partial pressure using an inspired fraction of oxygen of 1.0, Qs/Qt: right-to-left shunt fraction, OLV: one-lung ventilation, OLF: one-lung flooding, TLV: two-lung ventilation, CO group: control group, OLF group: OLF experimental group; *P < 0.05, significant compared with the CO group, ^#^*P* < 0.01, significant compared with baseline values, ^+^*P* < 0.01, significant compared with OLV/apnoea.
